# Neural decoding of expressive human movement from scalp electroencephalography (EEG)

**DOI:** 10.3389/fnhum.2014.00188

**Published:** 2014-04-08

**Authors:** Jesus G. Cruz-Garza, Zachery R. Hernandez, Sargoon Nepaul, Karen K. Bradley, Jose L. Contreras-Vidal

**Affiliations:** ^1^Laboratory for Noninvasive Brain-Machine Interface Systems, Department of Electrical and Computer Engineering, University of HoustonHouston, TX, USA; ^2^Center for Robotics and Intelligent Systems, Instituto Tecnológico y de Estudios Superiores de MonterreyMonterrey, Mexico; ^3^Department of Biomedical Engineering, University of HoustonHouston, TX, USA; ^4^Department of Neurobiology, University of Maryland, College ParkMD, USA; ^5^Department of Dance, University of Maryland, College ParkMD, USA

**Keywords:** EEG, neural classification, mobile neuroimaging, neural decoding, dance, Laban Movement Analysis

## Abstract

Although efforts to characterize human movement through electroencephalography (EEG) have revealed neural activities unique to limb control that can be used to infer movement kinematics, it is still unknown the extent to which EEG can be used to discern the expressive qualities that influence such movements. In this study we used EEG and inertial sensors to record brain activity and movement of five skilled and certified Laban Movement Analysis (LMA) dancers. Each dancer performed whole body movements of three Action types: movements devoid of expressive qualities (“Neutral”), non-expressive movements while thinking about specific expressive qualities (“Think”), and enacted expressive movements (“Do”). The expressive movement qualities that were used in the “Think” and “Do” actions consisted of a sequence of eight Laban Effort qualities as defined by LMA—a notation system and language for describing, visualizing, interpreting and documenting all varieties of human movement. We used delta band (0.2–4 Hz) EEG as input to a machine learning algorithm that computed locality-preserving Fisher's discriminant analysis (LFDA) for dimensionality reduction followed by Gaussian mixture models (GMMs) to decode the type of Action. We also trained our LFDA-GMM models to classify all the possible combinations of Action Type and Laban Effort quality (giving a total of 17 classes). Classification accuracy rates were 59.4 ± 0.6% for Action Type and 88.2 ± 0.7% for Laban Effort quality Type. Ancillary analyses of the potential relations between the EEG and movement kinematics of the dancer's body, indicated that motion-related artifacts did not significantly influence our classification results. In summary, this research demonstrates that EEG has valuable information about the expressive qualities of movement. These results may have applications for advancing the understanding of the neural basis of expressive movements and for the development of neuroprosthetics to restore movements.

## Introduction

In recent years, neural engineering approaches to understanding the neural basis of human movement using scalp electroencephalography (EEG) have uncovered dynamic cortical contributions to the initiation and control of human lower limb movements such as cycling (Jain et al., 2013); treadmill walking (Gwin et al., 2010, 2011; Presacco et al., 2011, 2012; Cheron et al., 2012; Petersen et al., 2012; Severens et al., 2012; Schneider et al., 2013), and even robotic assisted gait (Wagner et al., 2012; Kilicarslan et al., 2013). Most of these studies however have been limited to slow walking speeds and have been constrained by treadmills or the cycling or robotic devices used in the tasks, and have yet to examine more natural, and therefore less constrained, expressive movements. To address this important limitations, a mobile EEG-based brain imaging (MoBI) approach may be a valuable tool for recording and analyzing what the brain and the body do during the production of expressive movements, what the brain and the body experience, and what or how the brain self-organizes while movements of physical virtuosity are modified by expressive qualities that communicate emotional tone and texture—the basic language of human interactions. These expressive patterns are unique to each person, and we organize them in such particular ways that they become markers for our identities, even at great distances and from behind (Williams et al., 2008; Hodzic et al., 2009; Ramsey et al., 2011).

Interestingly, studies of the so-called human action observation network, comprised of ventral premotor cortex, inferior parietal lobe, and the superior temporal sulcus, have shown dissociable neural substrates for body motion and physical experience during the observation of dance (Cross et al., 2006, 2009). Orgs et al. (2008) reported modulation of event-related desynchronization (ERD) in alpha and beta bands between 7.5 and 25 Hz in accordance to a subject's dance expertise while viewing a dance movement. Tachibana et al. (2011) reported gradual increases in oxygenated-hemoglobin (oxy-Hb) levels using functional near-infrared spectroscopy (fNIRS) in the superior temporal gyrus during periods of increasing complexity of dance movement. While current neuroimaging research aims to recognize how the brain perceives dance, no study has described the various modes of expressive movements within a dance in relation to human scalp EEG activity. Thus, the current study focuses on extracting information about expressive movements performed during dance from non-invasive high-density scalp EEG.

The study emerged from many questions about the differences in neural engagement between functional and expressive movement in elite performers of movement; specifically, dance, and movement theatre. The questions are important, because dance has been studied primarily as elite athletic movement, located in the motor cortex. And yet, dancers train for years to express nuanced and complex qualities in order to tell a story, express an emotion, or locate a situation. Where do these various communicative messages, manifested in expressive movers, fire? Are they part of the motor functions, or are other aspects of cognition involved? The questions therefore became the basis of an emergent inquiry, using the high-density scalp EEG. Since no previous data on the differences between these two modalities of movement have been found, the study is nascent. As the investigators planned for the research, it became clear from the lack of any prior studies making these distinctions that we would be gathering baseline data and demonstrating feasibility for further studies.

Our study utilized expert analysts and performers of expressive movement, all trained in Laban Movement Analysis (LMA) (Laban, 1971; Bradley, 2009). LMA is composed of four major components: Body, Space, Effort, Shape, which make up the grammar for movement “sentences,” or phrases. In this study, we focus on the Effort component, which represents dynamic features of movement, specifically the shift of an inner attitude toward one or more of four factors: *Space* (attention or focus), *Weight* (impact, overcoming resistance), *Time* (pacing), and *Flow* (on-goingness). Each factor is a continuum between two extremes: (1) *Indulging in or favoring* the quality and (2) *Condensing* or fighting against the quality. Table 1 illustrates the Laban's Effort qualities, each factor's indulging and condensing element, respectively with textual descriptions and examples.

**Table 1:** **Effort factors and effort elements (Zhao, 2001; Bishko, 2007; Bradley, 2009)**.

**Effort**	**Element**	**Description**
Space		Attention to the surroundings. “Where.” Spatial orientation
	Indirect	All-round awareness, three–dimensionality of space, flexible Example: waving away bugs, scanning room for misplaced keys
	Direct	Straight, linear action, attention to singular spatial possibility Example: pointing to a particular spot, threading a needle
Flow		Amount of control. “How.” Feeling of how movement progresses
	Free	Uncontrolled, unable to stop in the course of movement Example: flinging a rock into a pond, waving wildly
	Bound	Rigid, controlled, restrained, resisting the flow Example: carrying an filled up of hot tea, moving in slow motion
Weight		Sensing, Intention. “What.” Attitude of movement
	Light	Buoyant, weightless, sensitive Example: dabbing paint on a canvas, movement of feather
	Strong	Powerful, bold, forceful, determined Example: punching, pushing, wringing a towel
Time		Intention, decision. “When.” Lack or sense of urgency
	Sustained	Leisurely, lingering Example: yawning, smelling the flowers
	Quick	Unexpected, surprising, urgent, fleeting Example: swatting a fly, grabbing child from path of danger

LMA differentiates between functional and expressive movement. Functional movement is perfunctory, task-oriented, non-expressive movement. It can be highly skill-based and technically complex, but it does not communicate an attitude or express an emotion. An example of functional movement might be cycling or treadmill walking; when such activities are primarily about the mechanics of executing the action. Expressive movement occurs through shifts in thoughts or intentions, and communicates something about the personal style of the mover. Human beings communicate in both verbal and nonverbal ways; the nonverbal expressive aspects of movement are “read” as indicators of our unique personalities and personal style. For example, movement analysts would describe individuals as “hyper” or “laid-back” based, in part, on their Effort patterns. Individuals might have recurring moments of a Strong, Direct stance. Others may demonstrate recurring moments of Quick, Free, Light gestures that accent a sparkly or lively presence. These expressive components of movement do not occur in isolated ways from the other aspects of movement analysis (Body, Space, and Shape), but rather, modify movement events. They are capable of a wide range of such modifications, and the complex patterns of expressiveness make up unique movement signatures. In this way, familiar people can be identified from even great distances, simply from their Effort qualities. Unfortunately, prior research investigating natural expressive movement has been limited to motion capture technology (Zhao and Badler, 2005; Bouchard and Badler, 2007). The markers that track the body in movement are tantalizingly close to being able to trace movement qualities, but have not yet achieved legibility of the shift into expressive movement. Thus, the goal of this study is two-fold: (1) Identify those efforts and individual differences in such qualities from brain activity recorded with scalp EEG, and (2) further develop MoBI approaches to the study of natural unconstrained expressive movement.

Certified Laban Movement Analysts were used as subjects because of the extensive training in distinguishing between categories of movement as both observers and performers. The five subjects were also teachers of LMA, and had extensive experience in demonstrating the differences and unique qualities of each feature of expressive movement to students of the work. One of the researchers (Bradley) is a Certified Laban Movement Analyst and has been teaching the material for 30 years. Such experienced subjects and researcher allowed for the identification (and labeling) of shifts in performance from functional to expressive moments.

## Materials and methods

### Experimental setup

#### Subjects

Five healthy Certified Movement Analysts (CMAs) proficient in the expressive components of LMA participated in the study after giving Informed Consent. All subjects were professional teachers and performers of movement; either dancers or movement-based actors. One man and four women were studied with ages ranging from 28–62 years. Data from subject 2 were discarded due to technical issues during the recording that resulted in missing data or data of bad quality.

#### Task

The study consisted of three-trial blocks where synchronized scalp EEG and whole-body kinematics data were recorded during a ~5 min unscripted and individualized dance performance. Each trial block consisted of three Action Types (“neutral,” “think,” “do”). During “neutral” action, subjects were directed to perform functional movements without any additional qualities of expression. This was followed by the “think” condition where subjects continued to perform functional movements, but now imagined a particular Laban Effort quality instructed by the experimenter. Lastly, subjects executed (i.e., enacted) the previously imagined expressive movement during the “do” condition. Dancers were instructed to begin and end each Laban Effort quality cued by the experimenter, a professional movement analyst, in addition to a monotone auditory trigger at the onset of each condition. The sequence of Laban Effort qualities varied from trial-to-trial as well as from subject-to-subject. Nonetheless, all efforts were arranged such that the *indulging* (*favored*) element was preceded by *condensing* element of the Laban Effort quality. As we were interested in inferring expressive qualities, all the “neutral” instances, which were devoid of willed expressiveness, were collapsed within a superset “neutral” leaving therefore a total of 17 distinct classes of expressive movements to infer from scalp EEG (“neutral” + “think” × 8 efforts + “do” × 8 efforts).

### Data acquisition and preprocessing

**Figure 1:**
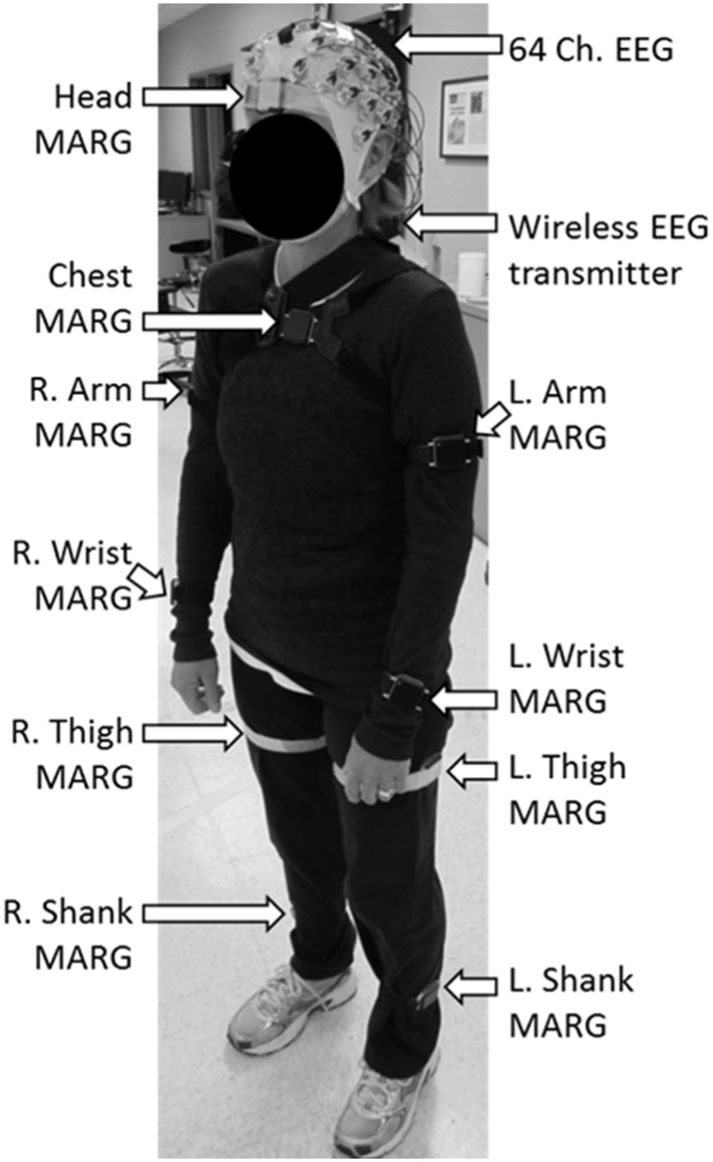
**Dancer wearing the 64 ch**. EEG cap and the 10 ch. magnetic, angular rate, and gravity (MARG) inertial sensors for data collection.

Brain activity was acquired non-invasively using a 64 channel, wireless, active EEG system sampled at 1000 Hz (BrainAmpDC with actiCAP, Brain Products GmbH). Electrode labeling was prepared in accordance to the 10–20 international system using FCz as reference and AFz as ground. The kinematics of each dance's movements were captured using 10 wireless Magnetic, Angular Rate, and Gravity (MARG) sensors (OPAL, APDM Inc., Portland, OR) sampled at 128 Hz mounted on the head, upper torso, lumbar region, arms, thighs, shanks, and feet. Each sensor contains a triaxial magnetometer, gyroscope, and accelerometer (Figure 1). A Kalman filter was used to estimate the orientation of each IMU with respect to the global reference frame. Using this information about sensor orientation, the tri-axial acceleration data, which had been compensated for gravitational effects, was estimated (Marins et al., 2001).

Peripheral EEG channels (FP1-2, AF7-8, F7-8, FT7-10, T7-8, TP7-10, P7-8, PO7-8, O1-2, Oz, PO9-10 in the extended 10–20 EEG system montage) were rejected as these channels are typically heavily corrupted with motion artifacts and scalp myoelectric (EMG) contamination. In addition, time samples of 500 ms before and after the onset of each condition were removed from further analysis to minimize time transition effects across conditions. EEG signals were resampled to 100 Hz, followed by a removal of low frequency trends and constrained to the delta band (0.2–4 Hz) using a 3rd order, zero-phase Butterworth band-pass filter. The EEG data were then standardized by channel by subtracting the mean and dividing by the standard deviation. Finally, a time-embedded feature matrix was constructed from *l* = 10 lags corresponding to a *w* = 100 ms window of EEG data. The embedded time interval was chosen based on previous studies demonstrating accurate decoding of movement kinematics from the fluctuations in the amplitude of low frequency EEG (Bradberry et al., 2010; Presacco et al., 2011, 2012). The feature vector for each time sample *t*_*n*_ was constructed by concatenating the 10 lags (*t*_*n*_ − 9, *t*_*n*_ − 8, …, *t*_*n*_) for each channel into a single vector of length 10 × *N*, where *N* is the number of EEG channels. To avoid the problem of missing data, the feature matrix was buffered by starting at the 10th EEG sample of the trial. All EEG channels and time lags were subsequently concatenated and standardized to form a [*t*_0_ − *w*] × [*N* * *l*] feature matrix.

### Dimensionality reduction

Once feature matrices were generated for all trial blocks, training and testing data were randomly sampled in equal sizes for each class for cross-validation purposes, and reduced in dimensionality (Bulea et al., 2013; Kilicarslan et al., 2013). Local Fisher's Discriminant Analysis (LFDA) is deployed here to reduce the dimensionality of a sample set of classes by minimizing and maximizing samples within and between classes, respectively, while preserving the locality of the samples that form each class (Sugiyama, 2006, 2007). Details of the technique adopted here (LFDA) are described in Sugiyama (2006, 2007).

### Neural classifier algorithm

A Gaussian mixture model (GMM), capable of representing arbitrary statistical distributions as a weighted summation of multiple Gaussian distributions, or components (Paalanen et al., 2006), was employed to classify the Laban Movement (LBM) Efforts from scalp EEG. As the name implies, GMM represents each class as a mixture of Gaussian components whose parameters and component number are approximated using the Estimation-Maximization (EM) algorithm and Bayes Information Criterion (BIC), respectively (Li et al., 2012). The two main parameters for this algorithm include the number of reduced dimensions *r* and *k*-nearest neighbors *k*_*nn*_ (from the LFDA) and thus must be optimized for this particular application of expressive movement classification (Li et al., 2012; Kilicarslan et al., 2013).

The probability density function for a given training data set *X* = {*x*_*i*_}^*n*^_*i* = 1_ ∈ ℝ^*d*^ is given by:

(1) $$p(x)=\sum_{k\;=\;1}^{K}\alpha_{k}\phi_{k}$$

(2) $$\phi_{k}(x)\;=\frac{e^{-0.5{\left(x\;-\;\mu_{k}\right)}^{\text{T}}\Sigma_{k}^{-1}(x\;-\;\mu_{k})}}{{\left(2\pi \right)}^{d/2}{\left|\Sigma_{k}\right|}^{1/2}}$$

where *K* is the number of components and α_*k*_ is the mixing weight, μ_*k*_ is the mean, and Σ_*k*_ is the covariance matrix of the *k*-th component. The parameters of each GMM component *K*, including α_*k*_, μ_*k*_, and Σ_*k*_, are estimated as those which maximize the log-likelihood of the training set given by:

(3) $$L_{k}=\sum_{i\;=\;1}^{n}\log p_{k}\left(x_{i}\right)$$

where *p*(*x*) is given in (1). Maximization of (3) is carried out using an iterative, greedy expectation-maximization (EM) algorithm (Vlassis and Likas, 2002), with the initial guess of the parameters α_*k*_, μ_*k*_, and Σ_*k*_ established via k-means clustering (Su and Dy, 2007), until the log-likelihood reaches a predetermined threshold. The determination of *K* is critical to successful implementation of GMMs for classification. The BIC has been reported as an effective metric for optimizing *K* (Li et al., 2012).

(4) $$BIC=-2L_{max}\;+\;2\log\left(n\right)$$

where *L_*max*_* is the maximum log-likelihood of each model from (3). During training, the maximum value of *K* = 10 was chosen based on estimates from prior work in our lab (Kilicarslan et al., 2013). We then computed *L*_*max*_ for each value of *K* ∈ {1, 2,…, 10} and estimated the optimal value of *K* as the model, using the minimum BIC from (4). In this manner, class-specific GMMs representing each Effort could be specified for use in a maximum-likelihood classifier. The parameters for each class-conditional GMM were specified using an optimization data set (classifier optimization). The posterior probability of each new data point was computed using the optimized model for each class, and that data point was then assigned to the class that returned the largest value.

Neural classification from scalp EEG was performed using two schemes of class initialization. We defined the Scheme 1 (Action Type) as a differentiation of *n* time samples into one of three classes corresponding to the conditions of “Neutral,” “Think,” and “Do.” In a similar initialization for Scheme 2 (Laban Effort quality Type), each condition of “Think” and “Do” were segregated into each of the eight Laban Effort quality elements, thereby forming an accumulation of 17 classes. The results of each classification could be observed by obtaining the confusion matrix of each classification scheme. This matrix provides the user with a detailed understanding of the overall accuracy rate in terms of the accuracy, or sensitivity and precision, for each class.

### Cross validation

**Figure 2:**
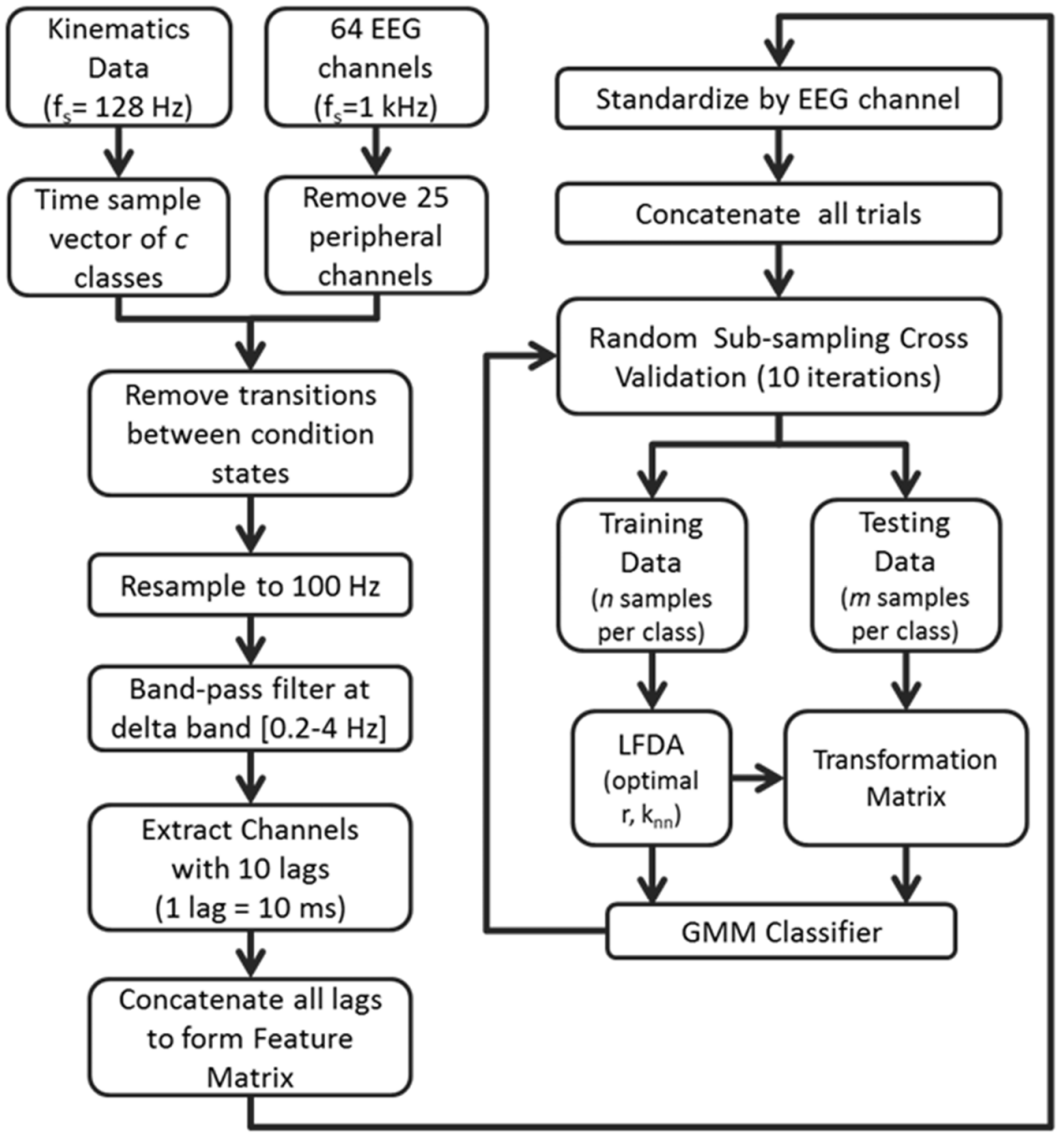
**Flow diagram depicting the computational approach to neural decoding of expressive movements**. The sample sizes *n* and *m* are equivalent to a percentage of the least populated class size.

Overall classification accuracy and class precision rates were averaged by implementing a random sub-sampling cross validation scheme. That is, samples from the concatenated feature matrix of three trial blocks were randomly selected and placed into an equal number of samples per class based on a percentage of samples from the least populated class. This process was then repeated 10 times (Figure 2) in order to minimize the effects of random sampling bias, avoid over-fitting, and demonstrate replicability of the algorithm. A sampling of 10 accuracies was found to be sufficient as it usually resulted in a low standard error (ε < 1).

### Forward selection of EEG channels

In an attempt to identify the EEG channels that contributed most to classification accuracy, the iterative process of forward selection was introduced upon the EEG channels and their corresponding lags that comprise the feature matrix. This was performed by computing the mean classification accuracy of each EEG channel independently using the LFDA-GMM algorithm, and ranking them in descending order of accuracy values. The highest ranked channel was added to the selected channels list (SCL), and tested against each of the remaining channels. The channel that ranked highest in classification accuracy when tested along the SCL was added to the SCL for the next iteration. This procedure was repeated until all remaining non-SCL channels were exhausted.

### Examination of potential mechanical artifacts on EEG decoding

To assess the potential contribution of mechanical/motion artifacts to decoding, we performed a series of analyses including time-frequency analysis, cross-correlation analysis, and coherence analysis to compare the EEG signals with the motion signals acquired with the MARG sensors. First, we performed principal component analysis (PCA; Duda et al., 2012) on the acceleration data (*d* = 10 sensors). A cross-correlation analysis was then performed between the raw EEG (resampled to 100 Hz) and the first “synergy” (i.e., first PC) of acceleration data. Histograms and box plots of each EEG channel by PC1 calculated correlation values were subsequently assessed to observe differences across the distribution of each class. Second, we performed a time-frequency analysis to compare the raw EEG signals over selected frontal, lateral, central, and posterior scalp sites and the gravity-compensated accelerometer readings from the MARG sensor placed on the head. Then, we estimated the coherence between the raw EEG signals and the accelerometer signals. Finally, we computed a whole-scalp cross-correlation of the EEG signals and the head accelerometer readings to examine the contribution of head motion to EEG.

## Results

### Kinematic analysis

**Figure 3:**
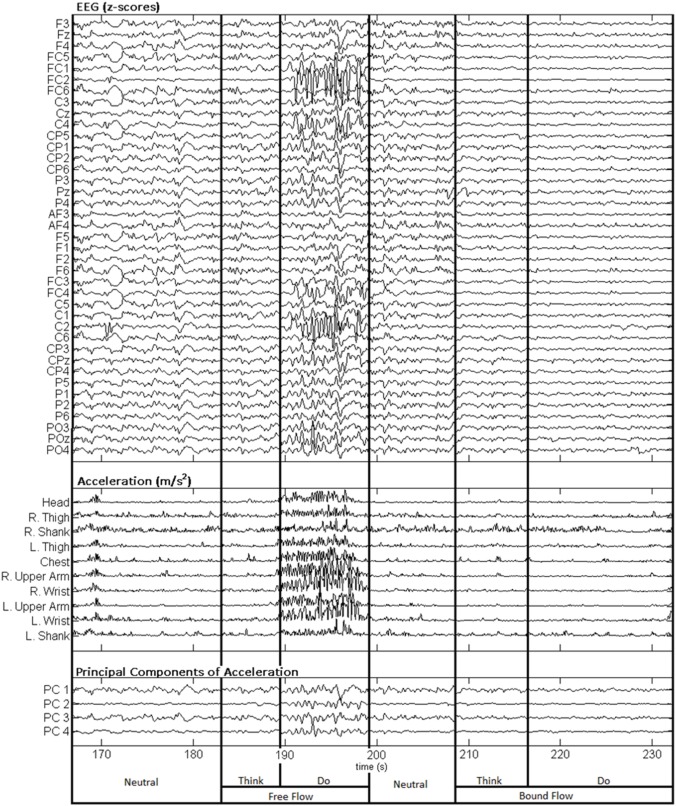
**Sample EEG and MARG recordings for Subject 4, Trial 2 with video recording (see Supplementary Materials)**. EEG and accelerometer data are segmented by each condition (Neutral, Think, Do) of the Laban Effort quality of Flow. The first four PCs of acceleration data are also shown.

**Figure 4:**
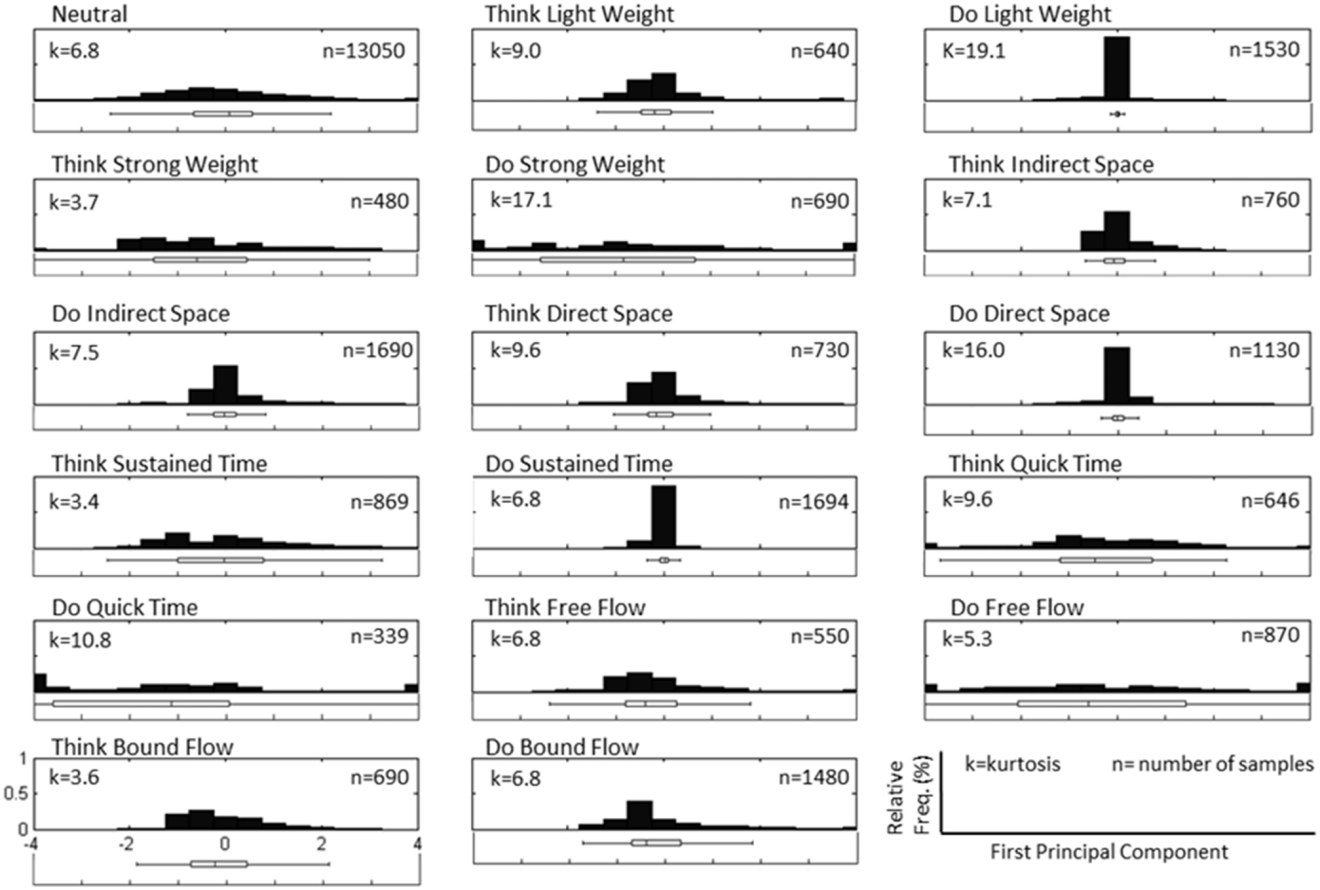
**Normalized histogram distribution of time sample data for the first principal component of magnitude acceleration data recorded from Subject 4, Trial 2 (*n* = number of samples, *k* = kurtosis)**. A boxplot representation excluding outliers of the distribution is shown below each histogram. Note that the distributions for PC1 are a combination of super-Gaussian and sub-Gaussian distributions as estimated by their kurtoses.

Figure 3 depicts a sample set of EEG and motion capture recordings for Subject 4, Trial 2 comprising all Action type classes for the Laban Effort quality of Flow, which includes the opposing elements of free and bound flows. PCA was performed upon the full time series of acceleration data from all 10 MARG sensors. The PCs whose cumulative variability summed to at least 80% were also featured within the sample set of signal data in Figure 3. Time series provided for both “neutral” blocks in Figure 3 appear to be relatively “smooth” (less varying) in terms of both neural activity and kinematic movement. One exception to this includes rapid changes in acceleration around 169 s as confirmed by the acceleration plots. EEG signal patterns are visually distinct between “think” time segments of free and bound flow elements, especially with unique areas of modulation of neural activity at 185 s (free flow) and 209 and 214 s (bound flow) which contained little to no effect of motion artifacts, as confirmed by the kinematics signal data. By contrast, the “do” section of the Laban Effort quality of free flow was found to contain the greatest influence of motion embedded in the EEG signal data, as demonstrated by the large excursions in signal magnitude for both EEG and kinematics data. These differences between classes are more prominent when the distribution of PC values can be observed for every class in the trial, as shown in Figure 4. Key features to note include the small variance accounted by “Do Light Weight” and “Do Sustained Time” classes, which reflects the low movement the subject effectuated for the particular action. Other classes such as “Do Free Flow” and “Do Quick Time” have a higher variance due to the nature of these efforts as they cover a greater range of motion. Potential motion artifacts produced by the subject's movements appear to contaminate EEG signal patterns, however the effect appears to be localized to specific classes of Laban Effort qualities (e.g., “Do Free Flow”) and thus not consistent over the entire time series. A more detailed analysis of potential mechanical/motion artifacts based on cross-correlation, coherence and time-frequency analyses are thus provided next.

**Figure 5:**
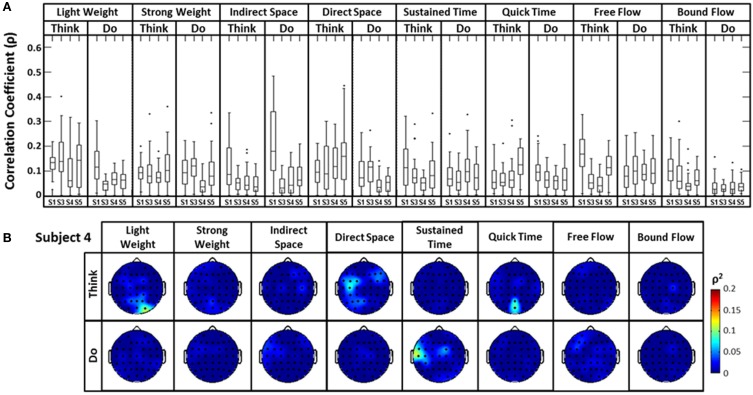
**The boxplots (A) and scalp maps (B) show the distribution of the cross-correlation coefficients and coefficients of determination between raw EEG signals and the first PC of the magnitude acceleration data across subjects and efforts**. The first PC of the acceleration data accounted for 64.5, 39.3, 59.8, and 44.9% of the variance for subjects 1, 3, 4, and 5 respectively. Asterisks (^*^) indicate outliers within the set of *ρ*-values.

The distribution of correlation values between raw EEG channels and the first PC of the raw acceleration data returned a range of median correlation coefficients between 0.02 and 0.15 across classes (Figure 5A). Outliers were identified for some efforts, and thus may be indicative of a close relationship between a particular EEG channel and the first PC “synergy” of acceleration. The coefficient of determination was obtained by squaring each correlation coefficient *ρ*. This coefficient is defined as the percent variation in the values of the dependent variable (raw EEG) that can be explained by variations in the values of the independent variable (acceleration). Coefficients of determination (*ρ*^2^) values were generally low and ranged from ~0.0 to ~0.23 (that is, ~0 to 23% of the total variation of the raw EEG can be accounted for by changes in the PC1) across all subjects and electrodes. Spatial distributions of *ρ*^2^-values were plotted as scalp maps to indicate the relationship between the raw EEG and the head acceleration across scalp channels. Peaks of highest accounted variance (Figure 5B) were observed for certain Laban Effort qualities, most notably in the occipital regions for “Think Quick Time” and “Think Light Weight” and temporal regions for “Do Sustained Time” for Subject 4 (See Supplementary Material for *ρ*^2^ data from other subjects).

**Figure 6:**
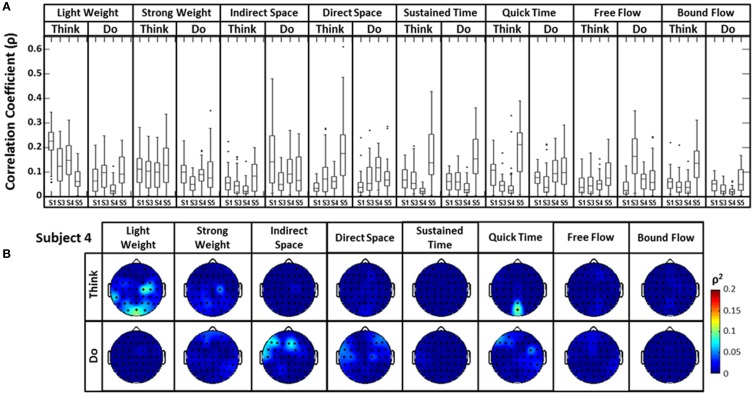
**(A)** The boxplots show the distribution of the cross-correlation coefficients between raw EEG signals and the magnitude acceleration data from the *head* MARG sensor across subjects and efforts. **(B)** Scalp maps of coefficient of determination (*ρ*^2^) values between raw EEG signals and the magnitude acceleration data from the head MARG sensor across Laban Effort qualities for Subject 4. See Supp. Materials for other subjects. Asterisks (^*^) indicate outliers within the set of *ρ*-values.

A similar analysis comparing the raw EEG signals and the head accelerometer (which directly recorded EEG electrode movements), rather than the first PC “synergy,” was also conducted (Figure 6). This resulted in correlation values generally below *ρ* = 0.15, though many boxplot distributions varied by subject throughout each Laban Effort quality (Figure 6A). Although strong relationships between the accelerometer and EEG signals may be expected, the relatively low *ρ*^2^ scores indicate otherwise. Low correlations between neural activity and head motion were observed for classes such as “Bound Flow,” which is reasonable given the rigid-like movements that this effort entails. In contrast, much higher correlation coefficients remained for “Light Weight” and “Indirect Space” time segments. Figure 6B depicts scalp maps with *ρ*^2^-values between head accelerometer and raw EEG data for Subject 4. In the scalp maps some classes show channels with slightly high correlation *ρ*^2^ = 0.1 (which account for ~10% of the total variation of the EEG due to the head motion), specifically in “Think Light Weight,” “Think Direct Space,” “Think Quick Time,” and “Do Sustained Time,” for Subject 4. Overall, these analyses showed a slight contamination, for some classes of Laban Effort qualities, of EEG signals due to head movement (see Supplementary Material), but the amount of total variance in the EEG signals explained by head motion was relatively small.

**Figure 7:**
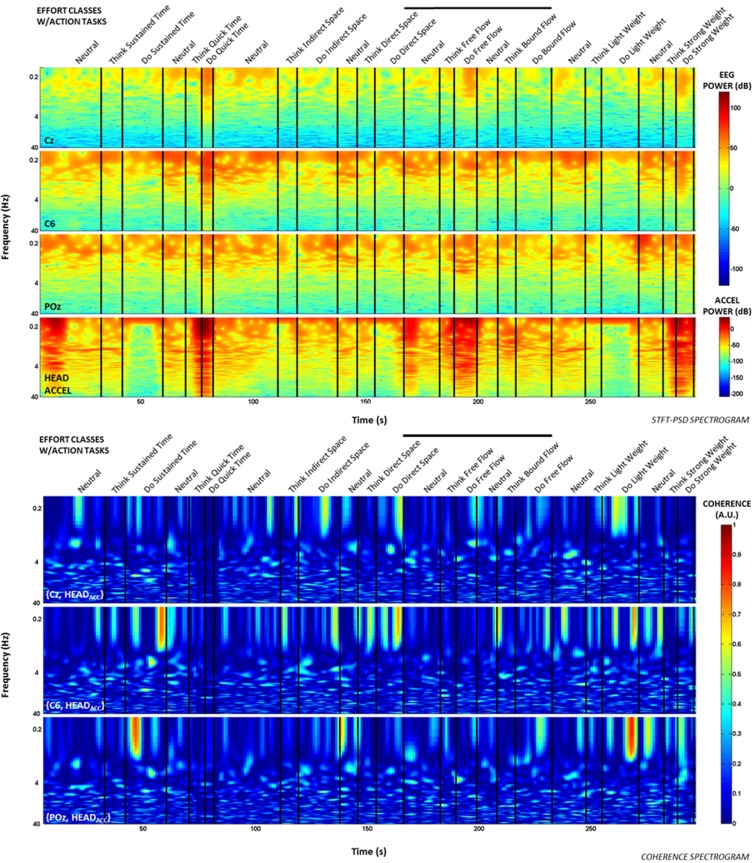
**Spectrograms and short-term coherence between selected (raw) EEG channels (Cz, C6, and POZ) and the acceleration magnitude of the head MARG sensor for Subject 4**. Frequency axes are shown in logarithmic scale. Note the generally low coherence values (<0.3) across most Laban Effort quality classes with some short-lived increases in coherence for some Efforts. Bold vertical black lines above each figure indicate the efforts windows in Figures 3, 4 to compare to each spectrogram plot.

Additionally, time-frequency and coherence analyses were performed upon the raw signals of three selected EEG electrodes (Cz, C6, and POz) representing a sampling of the spatial assortment of neural activity across the scalp, as well as the gravity-compensated acceleration magnitude of the head MARG sensor by generating two spectrograms, as shown in Figure 7. The spectrograms were generated by computing the short-time Fourier transform (STFT) over a time window of samples with overlap at each PSD computation of the FFT. We used a frequency range between 0.1–40 Hz and a time window of 1024 samples with 93% overlap. The mean-squared coherence between the head acceleration and each corresponding EEG electrode at each frequency value was computed using Welch's overlapped-segment averaging technique (Carter, 1987). From the spectrograms it can be observed that the actions “Do Quick Time,” “Do Think Free Flow,” “Do Strong Weight,” and short-lived portions of “Neutral” tasks contained higher power in the head accelerometer readings that may affect decoding. However, coherence estimates were generally low (<0.3; see Figure 7) with some transient increases in coherence between EEG and head acceleration during some Laban Effort qualities. Given that relatively high levels of coherence were short-lived and localized to a few classes of Laban Effort qualities, and that random sampling of EEG signals were used for training and cross-validation of our neural classifiers, we argue that motion artifacts, if present, had only a very minor contribution to decoding. We further discuss these results below.

### Decoding action type from scalp EEG

**Figure 8:**
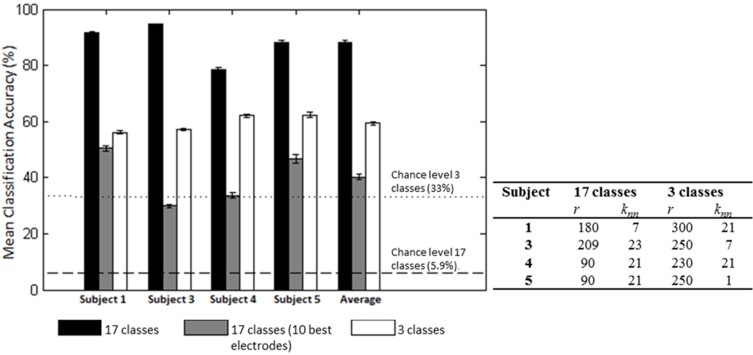
**Mean (*SD*) classification accuracies for 10 iterations and optimized LFDA parameters for both Action (3 classes) and Effort (17 classes) Type decoding**. The gray middle bars show the mean classification accuracy for the 10 electrodes that *individually* yielded the highest classification accuracy using the forward selection algorithm with constant LFDA parameters (*r* = 10, *k*_*nn*_ = 7, See Relevant EEG channels for classification for discussion).

We first examined the feasibility of inferring the action type (“neutral,” “think,” “do”), irrespective of Laban Effort quality, from scalp EEG. Analyses showed the “think” condition had the highest sensitivity than the other two action types. Based on the optimization of LFDA parameters, the mean accuracy rate (10 random subsampling cross-validation iterations were used for each subject) was 56.2 ± 0.6% by Action Type for Subject 1 (*r* = 300, *k*_*nn*_ = 21), which was well above 33% chance probability. Similar classification accuracy results were obtained for the rest of the subjects, namely 57.0 ± 0.4% for Subject 3, 62.1 ± 0.5% for Subject 4, 62.4 ± 1.0% for Subject 5. Figure 8 shows the mean classification accuracies for the different data sets tested.

**Figure 9:**
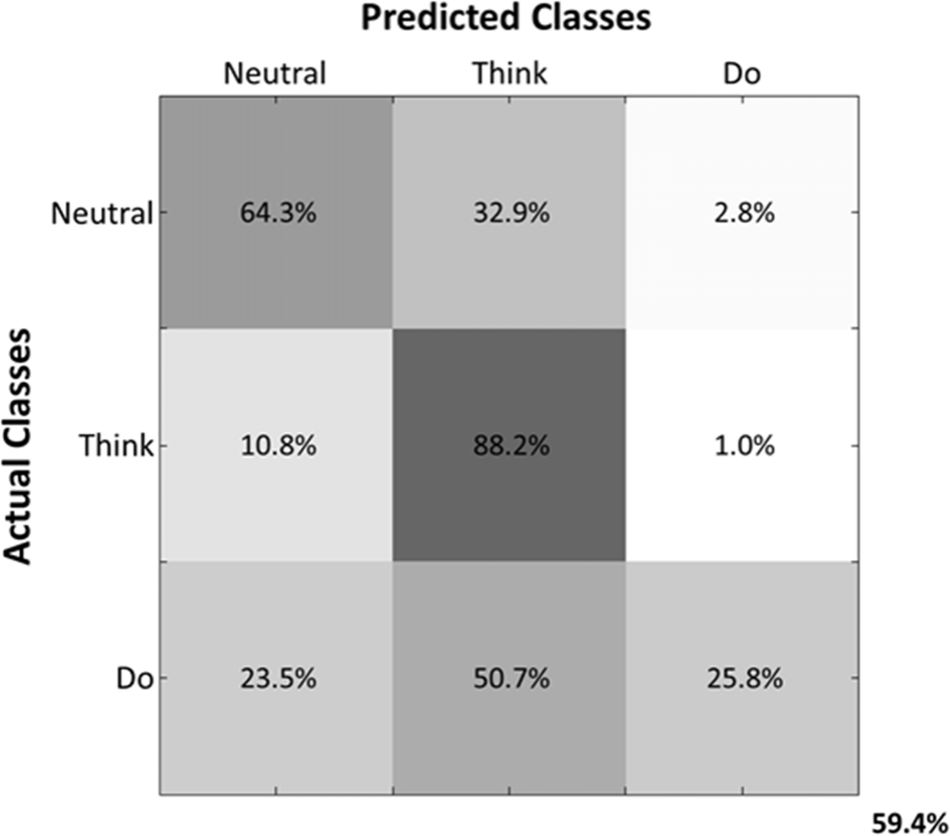
**Normalized Summed Confusion Matrix across subjects for three classes (Action Type decodes)**. The bottom-right corner provides the overall mean classification accuracy (59.4%).

Predicted samples were summed across all four subjects and normalized by dividing each predicted sample size by the actual class sample size, as indicated by the percentages within each confusion matrix block (Figure 9). Figure 9 depicts the confusion matrix for the Action Type decodes. Classification of EEG patterns corresponding to the “think” class achieved the highest classification rates (88.2%), followed by both “neutral” and “do” classes. Note that the highest misclassifications occurred for class “neutral,” which were classified as belonging to the “think” (32.9%) class. The worst performance was for the “do” class as instances of “neutral” (23.5%) and “think” (50.7%) were misclassified as “do.”

### Decoding laban effort quality type from scalp EEG

**Figure 10:**
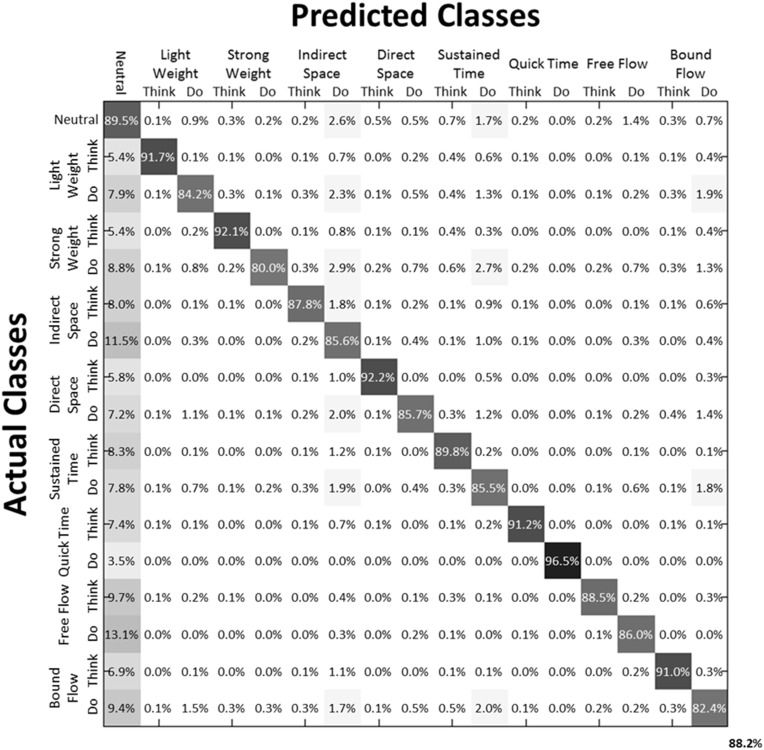
**Normalized Summed Confusion Matrix across subjects for 17 classes (Laban Effort quality Type)**. The bottom-right corner provides the overall mean classification accuracy across subjects (88.2%). This was obtained by summing each subject's normalized confusion matrix and normalizing the summed result.

We then examined the classification accuracy for Laban Effort quality Type (8 Think about Laban Effort quality + 8 Do Laban Effort quality + Neutral = 17 classes). In this case, nearly all test samples were accurately classified into their respective classes, which resulted in 88.2% classification accuracy across subjects. Figure 8 (black bars) shows the mean classification accuracies for Laban Effort qualities across subjects. Interestingly, most test samples were misclassified under the “neutral” class as shown by the relatively high percentages for all non-“neutral” classes in the first column (Figure 10). Based upon Figure 10, classes related to actions of “do” were more difficult to classify (relative to actions of “think”) except for “Do Quick Time,” which contained the highest sensitivity rate overall (96.5%).

### Training sample size effects on classification accuracy

**Figure 11:**
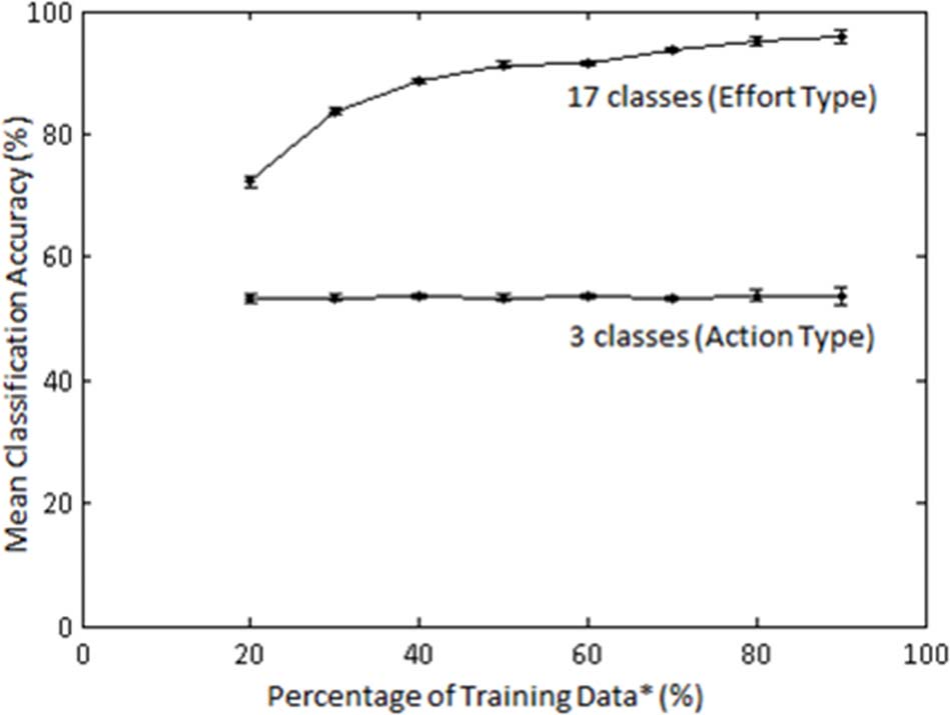
**Mean accuracies (for 10 iterations) across varying percentage of training samples for classification by Action (3 classes) and Laban Effort quality (17 classes) types for Subject 1**. LFDA parameters: (*r* = 180, *k*_*nn*_ = 7) for both classification schemes. ^*^Training data samples constitute a percentage of the least populated class.

The effect of training sample size on classification accuracy was also examined in Subject 1. The training sample size constituted a percentage (20–90) of the least populated class. Classification of Action type was not significantly affected by percentage of training samples (Figure 11); however, classification of Laban Effort quality type showed a non-linear increase as a function of percentage of training samples.

### Relevant EEG channels for classification

**Figure 12:**
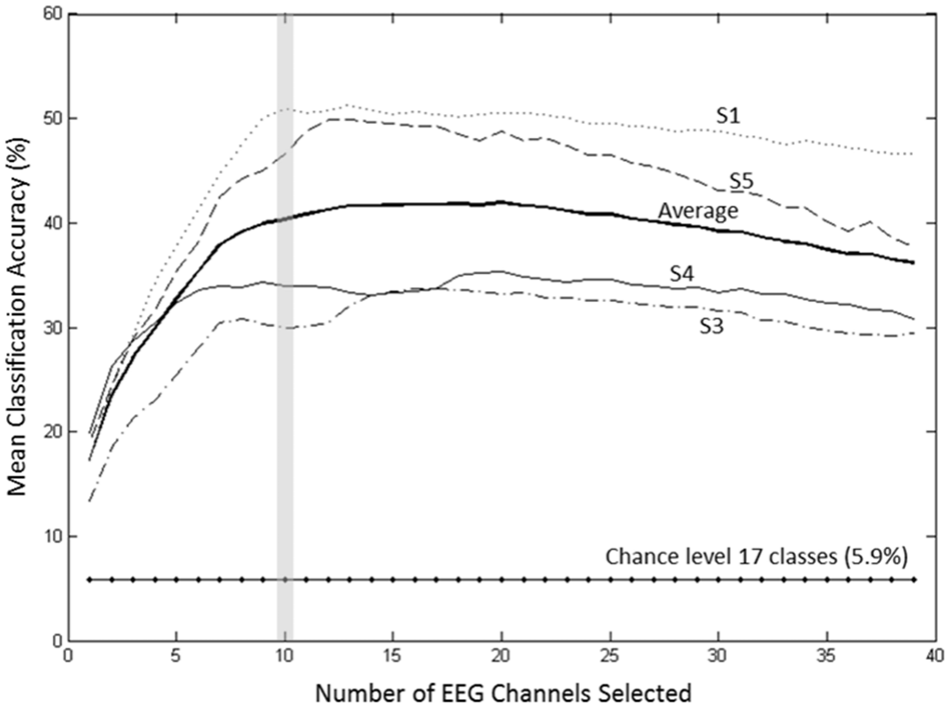
**Growth of the mean accuracy (for 30 iterations) as *n* channels were added to the new set for subjects 1, 3, 4, and 5 using forward selection with constant LDFA parameters (*r* = 10, *k*_*nn*_ = 7) and the Effort Type classification scheme**. The approximate peak in accuracy rate at 10 electrodes, highlighted by the vertical gray bar, was displayed in Figure 8 to demonstrate the extent of classifying using only 10 electrodes at such a relatively low dimensionality.

**Figure 13:**
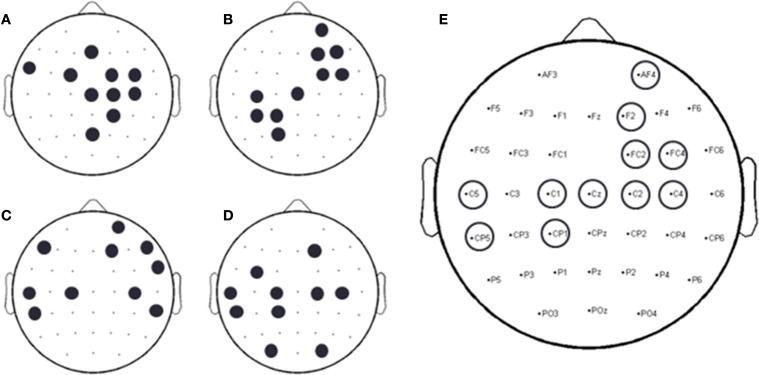
**Binary scalp maps for each subject depicting the first 10 electrodes identified as yielding the highest combined accuracy as computed by the forward selection algorithm. (A)** S1. **(B)** S3. **(C)** S4. **(D)** S5. **(E)** Electrodes common to a least two subjects, as indicated by the circles above a particular electrode channel. Given the subject-specific nature of neural decoding schemes, common, and unique neural patterns were expected (Lotte et al., 2007; Bradberry et al., 2010; Presacco et al., 2011, 2012; Wagner et al., 2012; Bulea et al., 2013).

A forward selection approach was employed per subject in order to identify the EEG channels with the most useful information for classification (Pagano and Gauvreau, 2000). While maintaining the number of reduced dimensions (*r*) and *k*-nearest neighbors (*k*_*nn*_) constant (*r* = 10, *k*_*nn*_ = 7) and operating under the Effort Type classification scheme, the mean classification accuracy was computed for all 39 channels and corresponding lags independently. The channel that yielded the highest classification accuracy (channel A) was then selected. Classification accuracies were then re-computed by adding channel A to each of the remaining 38 channels independently. The channel-pair yielding the highest accuracy was again selected and added to each of the remained channels to find the channel-triplets yielding the highest accuracy, and so on. This process continues until no channels remained, and classification accuracy was shown to stop increasing after selecting approximately 10 electrodes for each subject (shaded gray region in Figure 12). Hence, 10 electrodes were retained for further analysis per subject, as illustrated by the scalp maps depicted in Figures 13A–D. Electrodes common to at least two subjects were highlighted in Figure 13E, which span over scalp areas above bilateral premotor and motor cortices and dorsal parietal lobule areas. This is consistent with previous studies seeking to associate dancing movements with cortical regions (Cross et al., 2006, 2009). Though peak accuracies at 10 electrodes (Figure 12) were low (40–50%) relative to optimized Effort Type accuracies (Figure 8), this was largely due to the lower reduced dimension parameter for LDFA. This suggests that a higher-than-chance classification accuracy can be obtained by using as few as 10 electrodes. Nevertheless, relevant information within all 39 EEG channels ultimately allows the classifier to reach more than 90% decoding accuracy (Figure 8).

### Effects of head motion on neural classification

**Figure 14:**
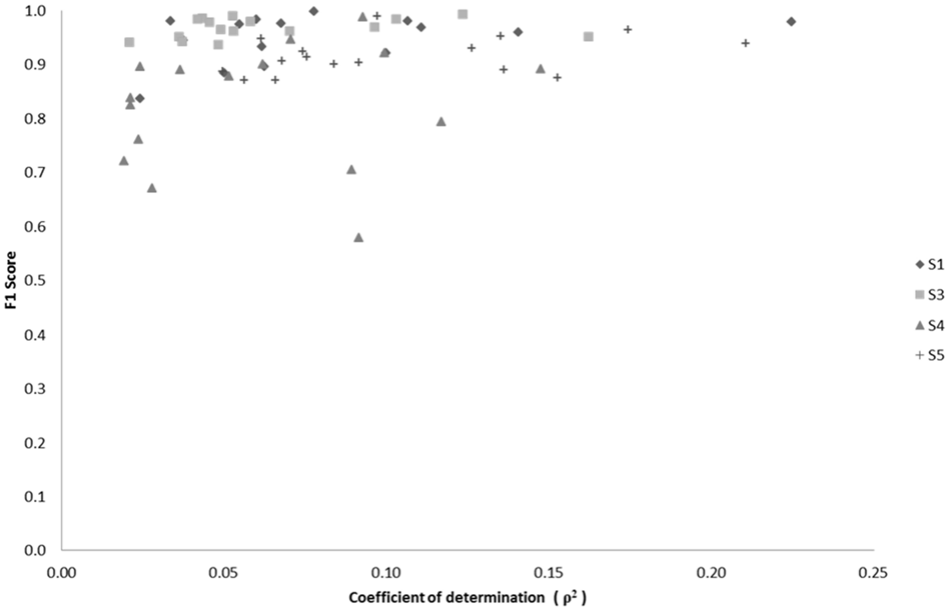
**Scatter plot of F1 score vs. median correlation for each of the 17 possible classes (Effort Types)**. The F1 score represents the weighted average between the precision and sensitivity rates of each class.

We examined the relationship between classification performance and motion artifact contamination. Taking the *ρ*-values from Figure 5A, we compared them with each class' F1 score in classification. If classes with higher *ρ*-values showed a higher F1 score, this would mean that the classifier was able to better classify the classes that were modulated by motion artifacts. However, Figure 14 shows no evidence of a correspondence between the F1 score and the correlation coefficients per class.

The F1 score (5) is a weighted average of the sensitivity and precision rates, and thus reflects the overall accuracy of a particular class (Hripcsak and Rothschild, 2005). For purposes of this study we use the balanced F1 score equation, defined as:

(5) $$F=\;\frac{\left(1+\beta^{2}\right)\times sensitivity\times precision}{\left(\beta \times precision\right)+sensitivity},\;\beta =1$$

where β is used as a weighting factor between sensitivity and precision. Overall, a direct relationship between classification success and the median correlation coefficient of EEG channels-to-acceleration data does not seem to occur, but rather a tendency exists for high successes of neural classification in classes that also contain low correlations with accelerometer data.

### Effort type classification represented in 4D laban space

**Figure 15:**
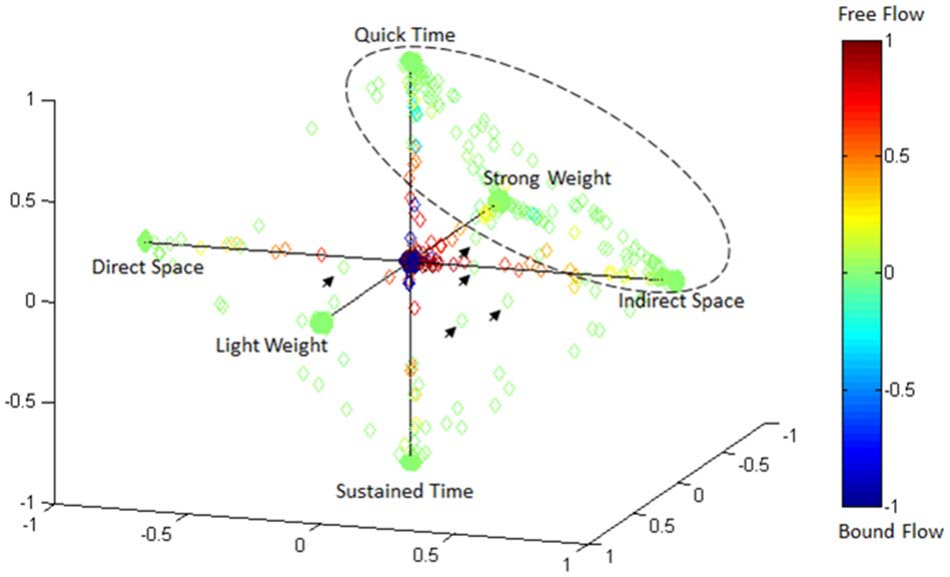
**Visualization of classification results for delta-band processed EEG data from “think” actions in 4D space of Laban Effort qualities**. Classification data from Subject 1, trials 1–3 are shown. Test samples were classified using the LFDA-GMM algorithm (*r* = 70, *k*_*nn*_ = 7, using training samples per class that constitute 50% of the least populated class). Decisions of correspondence between Laban Effort qualities were made using a probability density function of the output of the GMM. The respective probabilities were used as weightings for the four coordinates in this space. Clusters of data in the extremes of the octahedron for each Laban Element-Factor are clearly visible, while some samples remain not clearly distinguishable as pertaining to a specific class.

Figure 15 illustrates the highly predictive power of the Laban Effort quality Type neural classification scheme. Using a normalized variant of the GMM probability density function, we placed weightings to the four coordinates in the Laban Effort quality space. Each axis corresponds to a Laban Effort quality of *Space*, *Flow*, *Weight*, and *Time*. Some testing samples were found to be misclassified between *Indirect Space*, *Light Weight*, and *Quick Time* axes, as shown by the ellipsis in Figure 15. This may suggest shared characteristics between the expressive movements that cause such misclassification. Non-expressive, or non-classifiable, samples are depicted as green foci falling near the center of the plot, as indicated by the small arrows. The small amount of non-classified samples reflects the overall error of the classifier to predict Laban Effort quality using neural recordings.

## Discussion

### Classification of expressive movements from scalp EEG

In this study we demonstrate the feasibility of classifying expressive movement from delta band, EEG signals. Classification rates ranged from 59.4 ± 0.6% for decoding of Action Type (“neutral,” “think,” and “do”) to 88.2 ± 0.7% for decoding of Laban Effort quality (17 classes). Surprisingly, only the “think” class was reliably decoded from EEG whereas classes “neutral” and “do” were poorly decoded. It should be noted that subjects were not instructed to perform a particular pattern of movement, but rather a mode of action (“neutral,” “think,” and “do”) and Laban Effort quality as a component of LMA. Thus, subjects performed highly individualized changing movement patterns throughout the recording session irrespective of mode of action. We note that our neural decoding framework uses a within-subject approach where neural classifiers are trained for each subject. Such neural decoding approaches are subject specific (Lotte et al., 2007; Bradberry et al., 2010; Presacco et al., 2011, 2012; Wagner et al., 2012; Bulea et al., 2013), and thus common and unique neural patterns are to be expected to influence classification. Conventional statistical analyses can therefore be difficult to interpret in the context of this framework because many factors affect the resulting estimates of significance (i.e., assumptions underlying response distribution, sample size, number of trials, data over-fitting, etc.) (Tsuchiya et al., 2008). Given the cross-validation procedure (i.e., separate random sampling of data for training and test trials) used in our methodology, the risk of over-fitting is minimized. By deploying our methodology for investigating differences in cortical EEG activity patterns, especially as a function of within-subject training, valuable information could be learned about the adaptation/learning trajectories of those patterns and their relationship to performance and training. On the other hand, the consistency of the underlying neural representations, within a subject, would be a valuable metric in longitudinal studies.

### Decoding of action type and laban effort qualities

The mean decoding accuracy for action type (“neutral,” “think,” “do”) was near 60%, which was well above chance level. Interestingly, classification rate for the “think” actions was highest (88.2%), followed by “neutral” (64.3%) and “do” actions (25.8%). We note that individualized and unscripted functional movements were performed across all the three action types. Thus, the lowest classification rate for the “do” actions may reflect neural patterns that contain integrated elements of “thought” expressiveness and functional movement that were enacted by the dancers. This would have likely introduced “noise” to these patterns as diverse functional movements were performed irrespective of the Laban Effort qualities being imagined. On the other hand, the “neutral” actions, albeit unscripted and varying across time, contained separable information for the classifiers to discriminate them from the other action types. Only the “think” actions contained separable information about functional movement and Laban Effort qualities, which could be decoded by the classifiers. Thus, it is expected the “neutral” class to yield the worst classification rate given the stochastic pattern of functional movements it contains. Likewise the poor classification of the “do” class may be due to the heterogeneous mixture of functional and expressive movements co-occurring, which may introduce some neural noise within the neural activity evoked by this action.

Interestingly our results demonstrate a greater predictive power toward the classification of each Laban Effort quality element rather than the aggregation of all Laban elements into a singular condition-defined class (Figures 9, 10) suggesting that the neural internal states associated with these efforts contain differentiable features, beyond the movements performed, that can be extracted from scalp EEG.

### Influence of motion artifacts

Given the nature of the experimental setup, it is reasonable to assert the assumption that the EEG data may be plagued with motion artifacts. To examine this possibility we performed a series of analyses to uncover any potential relationship between the EEG signals and the dancers' body and head movements. We found that in a few instances the correlation between the raw EEG and the dancers' movements assessed via the MARG sensors was moderately high; however these effects appear to be localized to particular segments of time (see Figures 3, 4). We also note that periods of intense unscripted and varying functional movements may have been responsible for the periods of higher correlation and coherence estimates. However, we hypothesize that for the same reason, neural activity related to the “thinking” of Laban Effort qualities may have occurred or modulated varying body and head movements, thus making these motions likely irrelevant for classifiers. Additionally, the relatively low coefficients of determination between EEG and kinematics data demonstrated that the % variability of EEG signals accounting for head motion was rather small. Moreover, the random sampling of both training and testing datasets would have precluded the introduction of kinematic influences in both calibration and testing of the classifier, as the temporal nature of kinematic artifacts would have not been included in the training or testing data. This however warrants further investigation to develop better strategies of implementing MoBI approaches to capture neural mechanisms behind general movements.

Overall, our results show the feasibility of inferring the expressive component of movements (according to the Laban categorization) from scalp EEG, especially when those components are imagined as subjects perform unscripted natural body movements. These results may have implications for the study of movement training, disease and brain-computer interfaces for restoration of expressive movements.

### Conflict of interest statement

The authors declare that the research was conducted in the absence of any commercial or financial relationships that could be construed as a potential conflict of interest.

## Supplementary material

Video Recordings to Figure 3 **Subject 4, Trial 2 EEG and kinematics data**. Recordings are included in the online manuscript submission and are titled as: Direct Space Effort (Movie 1), Free Flow Effort (Movie 2), and Bound Flow Effort (Movie 3).

Figure 5.S1 **Mapping of the coefficient of determination (*ρ*^2^) between the first principal component of the accelerometer data and unprocessed EEG data for each Laban Effort quality performed by each subject**.

Figure 6.S1 **Mapping of the coefficient of determination (*ρ*^2^) between the head accelerometer magnitude and unprocessed EEG data for each Laban Effort quality performed by each subject**.
