# Supplementary material for: Neural decoding of expressive human movement from scalp electroencephalography (EEG)
Source: Front Hum Neurosci. 2014 Apr 8;8:188. doi: 10.3389/fnhum.2014.00188 (PMC3986521; doi:10.3389/fnhum.2014.00188)
Supplement: Figure 5.S1 — Mapping of the coefficient of determination (ρ2) between the first principal component of the accelerometer data and unprocessed EEG data for each Laban Effort quality performed by each subject. [file Presentation1.PDF]

1. Supplemental Materials

***Video Recordings to Figure 3:* Subject 4, Trial 2 EEG and kinematics data. Recordings are included in the online manuscript submission and are titled as: Direct Space Effort (Movie 1), Free Flow Effort (Movie 2), and Bound Flow Effort (Movie 3).**

Figure 5 Supplementary Material.

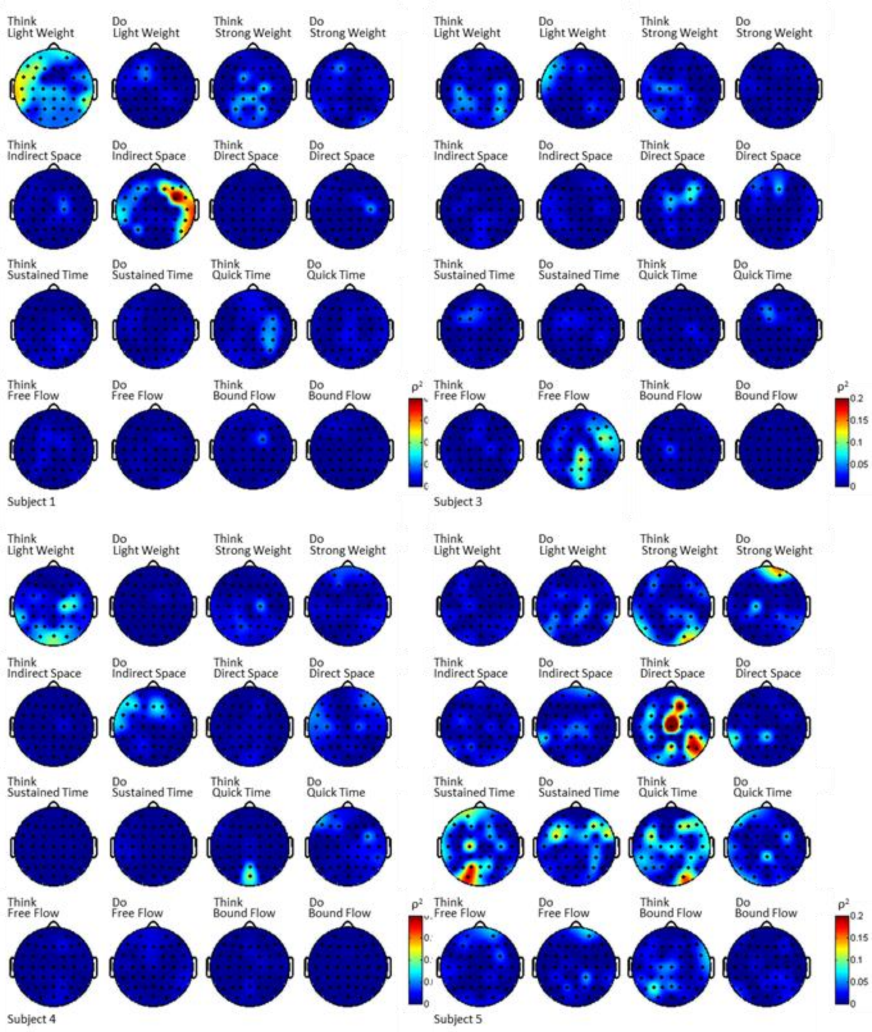

Figure 5.S1. Mapping of the coefficient of determination (ρ^2) between the first principal component of the accelerometer data and unprocessed EEG data for each Laban Effort performed by each subject.

Figure 6. Supplementary material.

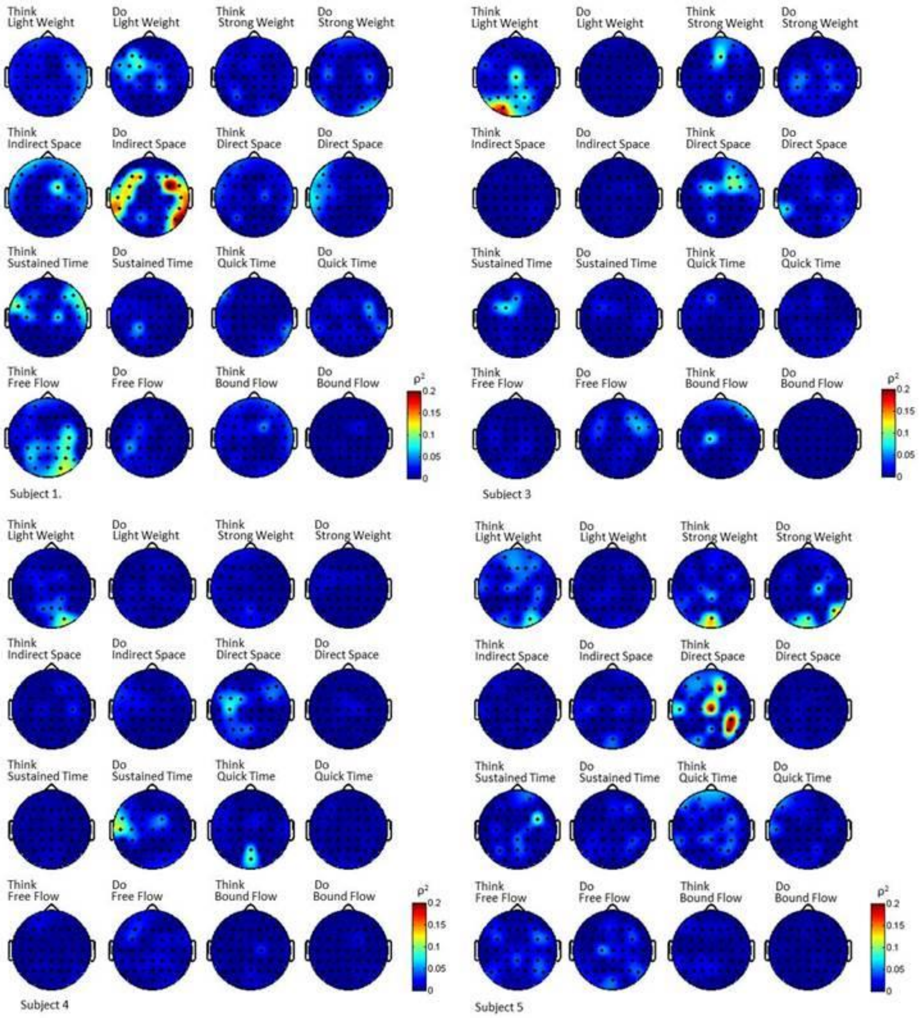

Figure 6.S1. Mapping of the coefficient of determination (ρ^2) between the head accelerometer magnitude and unprocessed EEG data for each Laban Effort performed by each subject.
